# Ruthenium-106 brachytherapy and central uveal melanoma

**DOI:** 10.1007/s10792-024-03381-6

**Published:** 2025-01-11

**Authors:** Luise Grajewski, Christiane Kneifel, Markus Wösle, Ilja F. Ciernik, Lothar Krause

**Affiliations:** 1 Department of Ophthalmology, Staedtisches Klinikum Dessau, Brandenburg Medical School Theodor Fontane, Dessau, Germany; 2 Department of Radiation Oncology, Staedtisches Klinikum Dessau, Brandenburg Medical School Theodor Fontane, Dessau, Germany

**Keywords:** Uveal melanoma, Brachytherapy, Central, Ruthenium

## Abstract

**Purpose:**

Uveal melanoma (UM) is the most common primary ocular malignancy. The size and location of the tumor are decisive for brachytherapy with the β-emitting ruthenium-106 (Ru-106) plaque. The treatment of juxtapapillary and juxtafoveolar UM may be challenging because of the proximity or involvement of the macula and optic nerve and high recurrence rates.

**Methods:**

Central UMs were defined as lesions up to 5 mm off the optic disc or fovea radius of 5 mm. Between January 2011 and July 2020, we treated 56 patients with Ru-106-brachytherapy. The clinical outcomes for recurrence, visual acuity, and radiation-related toxicity were assessed. The follow-up was 66 (6–136) months.

**Results:**

Of the 56 patients (56 eyes), 8 (14%) suffered from local recurrence. Six relapsing UM in 19 (32%) patients were located close to the optic disc, and two patients had UM close to the macula (2/37, 5%) (*p* > 0.05). The overall eye-preservation rate was 89%. The pretreatment visual acuity (VA) was 0.45 and reduced to 0.26 after brachytherapy. Radiation retinopathy or optic neuropathy was detected in 7 (13%) patients and radiation maculopathy in 10 (17.9%). Six patients (11%) underwent enucleation for recurrence or radiation-induced ophthalmopathy.

**Conclusion:**

Central UMs are challenging to treat. UMs should be categorized as lesions laterally or medially to the fovea because of different long-term control rates. Localization near the optic disc requires thoughtful management.

## Introduction

Uveal melanomas (UMs) are the most common primary ocular malignancies with an incidence in Europe of 1:100,000 annually [20]. Prognosis is determined by histology, genetic profile, base diameter, prominence, location, and ciliary body involvement. Every millimeter increase in apical height leads to a 5% increased risk for metastasis [22]. Distant metastases occur in up to 25% of patients after 5 years [6, 24].

Depending on the size and location of the tumor in relation to the optic nerve, macula, and ciliary body, different treatment options are available. In addition to brachytherapy in the form of contact therapy with ruthenium-106 (Ru-106) or iodine-125 (I-125), external beam radiation therapy options include proton beam therapy and stereotactic radiotherapy with photons [5]. We prefer brachytherapy for peripheral UMs or central UMs close to the fovea. In our practice, we use stereotactic radiotherapy with photons successfully for large UMs with an apical height of > 6 mm and UMs close to or abutting the optic disc and achieved high local control rates [26]. Regarding the situation of each patient such as older age, underlying diseases, and immobilization, different treatment options for personalized therapies such as Ru-106 brachytherapy with adjuvant transpupillary thermotherapy are also possible.

Brachytherapy with β-emitting Ru-106 plaque is an effective and widely used treatment option, which was first introduced by Lommatzsch and Vollmar [13]. The beta rays from the radiation shield ensure easy handling and optimal tumor dose coverage limited by the height of the UM for Ru-106 [7, 27]. Studies have reported successful treatment with Ru-106 plaque for UMs with an apical height of > 8 mm [11, 15].

UMs close to the macula and optic nerve are challenging to treat because the central presentation is a risk factor for local treatment failure. Although the main treatment goal is to control the UM and reduce the metastasis risk, precise placement of the plaque is key to minimizing the risk of damage to central sensitive structures through radiation complications, for example, radiation retinopathy and optic neuropathy, toxic tumor syndrome, and cataract and maintain good visual acuity (VA).

Here we present our results of Ru-106 brachytherapy for successful treatment of central UMs.

## Patients and methods

From January 2011 to December 2019, a total of 283 UMs were referred to our institution. Ru-106 brachytherapy was used in 171 patients, 3 eyes were enucleated, and fractionated stereotactic radiosurgery was performed in 109 patients.

**Fig. 1:**
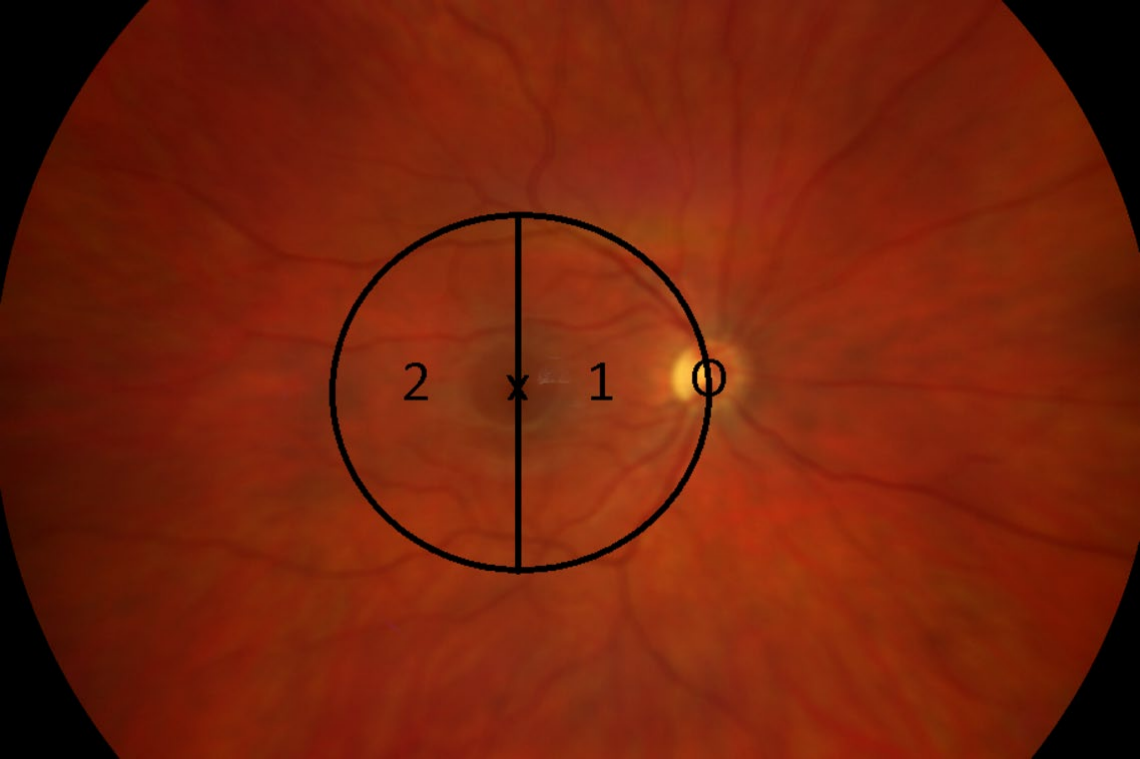
Categorization of tumor presentation according to the location in relation to the fovea. The fundus of a right eye: 1, group I; 2, group II; X, fovea; O, optic disc

In this retrospective cohort study, patients treated with Ru-106 brachytherapy and UM touching the macula were included. A total of 56 eyes of 56 patients with central UMs were analyzed. Central UMs were defined as juxtafoveolar UMs with a posterior edge abutting the optic disc–fovea radius around the fovea by 5 mm. The cases were divided in two groups. Group I included patients (19 eyes, 34%) with UM reaching the nasal half of the 5 mm discoid area around the fovea. Group II consisted of patients (37 eyes, 66%) with an UM localized on the temporal half of the foveal disc of 5 mm. If an UM touched both sides of the fovea, it was classified as group I (Fig. 1).

The diagnosis was made based on the clinical findings using ultrasonography. In addition, in a minority of cases, the diagnosis was confirmed using fluorescence angiography or magnetic resonance imaging studies. Malignancies were diagnosed based on the presence of orange pigment, exudative changes such as an accompanying detachment, or a documented tumor growth.

A Ru-106 plaque CCA with a diameter of 15.3 mm was used in 9 cases, a Ru-106 plaque CCB with a diameter of 20.2 mm in 16 cases, and a Ru-106 plaque CCD with a diameter of 17.9 mm in 31 cases (Eckert & Ziegler, BEBIG®, Berlin, Germany). After the circular opening of the conjunctiva and looping of the eye muscles, the external borders of the UM were marked on the sclera either by diaphanoscopy or binocular funduscopy. If it was necessary for the right plaque placement, extraocular muscles, straight eye muscles, and oblique muscles were disinserted at the time of treatment. The plaque was fixed onto the sclera with supramid 6–0, and right placement was controlled by binocular funduscopy with an indentation. If it was necessary to preserve the sensitive structures we placed the plaque. A dosage of approximately 110 Gy at the apex of the tumor was calculated. The plaque was removed.

The mean apical height was 3.9 (0.9–8.28) mm, 4.39 mm in group I, and 3.67 mm in group II. Nine eyes had an apical height of > 6 mm. The mean maximum diameter was 10.8 (5–16.8) mm; in group I, it was 11 mm, and in group II, it was 10.7 mm. According to the 8th AJCC classification, 25 UM were classified as T1, 23 UM as T2, and 8 UM as T3 (group I, T1, *n* = 7; T2, *n* = 8; T3, *n* = 4; group II, T1, *n* = 18; T2, *n* = 15; T3, *n* = 4).

In this study, 36 UMs (64%) had an exudative retinal detachment before brachytherapy, including 15 in group I (79%) and 21 in group II (57%). A CCD plaque was used in 31, a CCB plaque in 16, and a CCA plaque in 9 cases. The mean scleral dosage was 480 (200–1000) Gy, and the mean dosage at the apex was 105 (84–105) Gy.

After brachytherapy, patients were referred back to their local ophthalmologist for close follow-ups and were reviewed in our institute after 3 and 9 months and then yearly after brachytherapy or in closer intervals if needed. At each follow-up, VA, clinical examination, sonography, and fundus photography were performed. Metastasis surveillance by liver ultrasonography was performed every 6 months.

This retrospective review of patient data did not require ethical approval in accordance with the national guidelines. Written informed consent was obtained from the patients, before conducting the clinical examinations described above.

The U test of Mann, Whitney, and Wilcoxon was applied for univariate hypothesis testing with two independent samples. The time-dependent probabilities for local progression-free survival were determined by means of the Kaplan–Meier method. For the comparison of survival periods in two independent samples, the log-rank test based on χ2 was performed. Cox proportional hazards models were constructed to assess the significance of predictor variables by regressions. A significance level of α = 0.05 was used and all confidence levels were 1 – α = 0.95.

≙ 95%. A *p* value of ≤ 0.05 was considered to be significant. The graphical representation of the results, the statistical hypothesis testing, and the Cox proportional hazards regressions were performed by MATLAB®, version R2023b (The MathWorks, Inc., Natick, MA, USA).

## Results

**Fig. 2:**
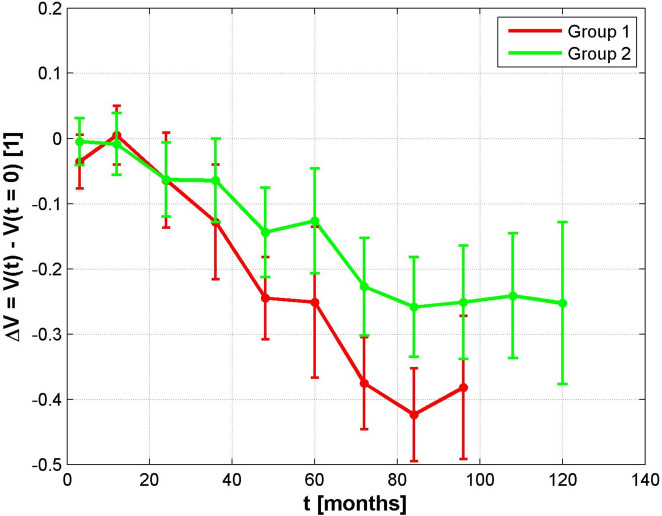
Change in visual acuity in groups I and II. The mean values are shown with the “standard error of the mean” dispersion measure

In this retrospective case series, 56 eyes of 56 patients (29 women, 27 men) with central UM, 24 (42%) right eyes, and 32 (58%) left eyes were included. The mean age was 65 (40–89) years. The mean follow-up time was 66 (6–136) months. The mean presenting decimal VA was 0.45 (group I, 0.37; group II, 0.49), and the final VA was 0.26 (group I, 0.15; group II, 0.32, *p* = 0.013) (Fig. 2).

**Fig. 3:**
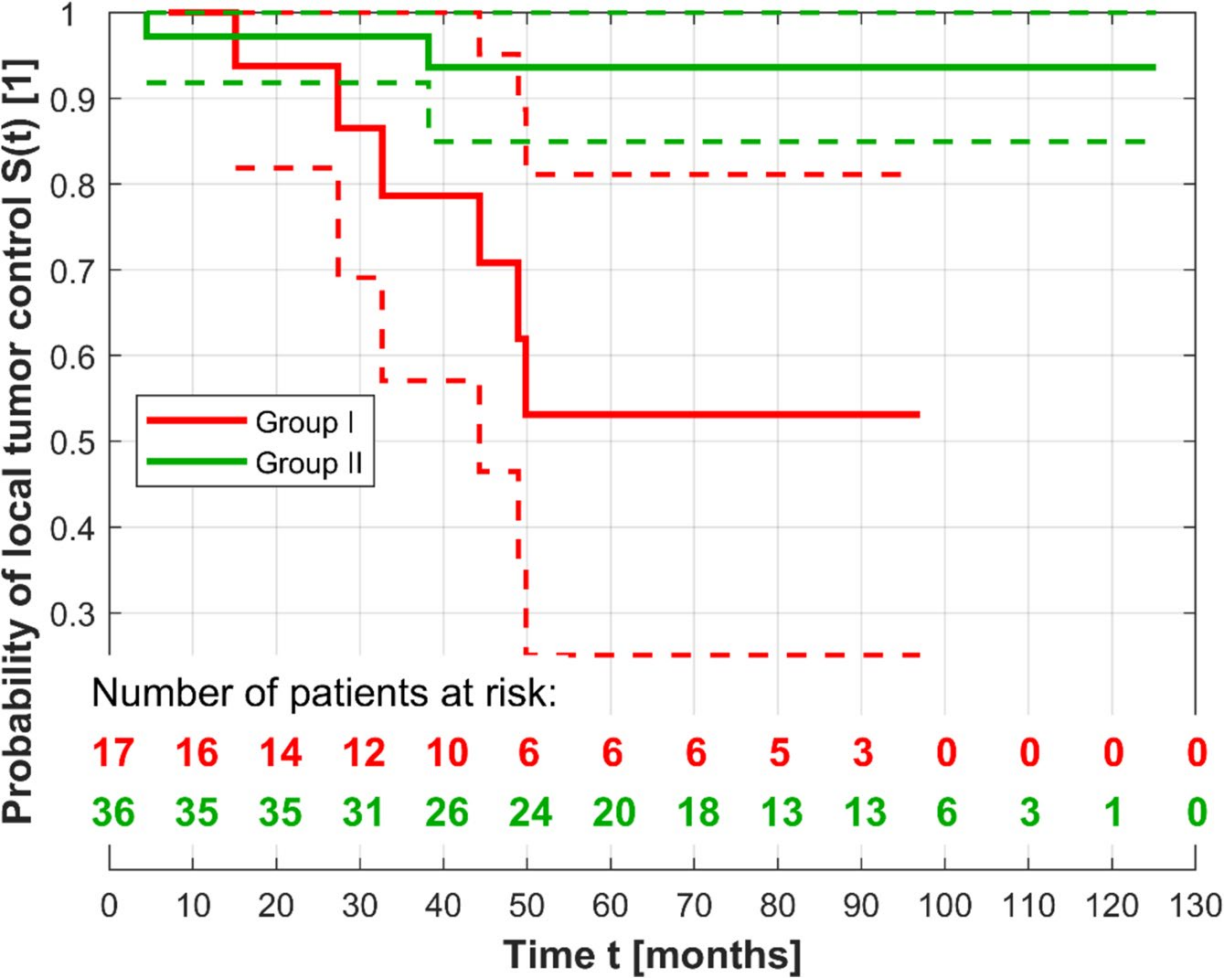
Kaplan–Meier curves (solid lines) of fully observed and right-censored data with lower and upper confidence bounds (dashed lines) for the Groups I and II

**Fig. 4:**
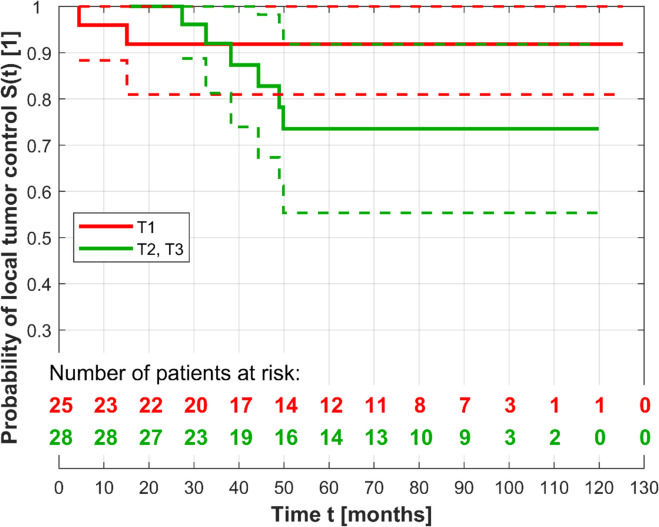
Kaplan–Meier curves (solid lines) of fully observed and right-censored data with lower and upper confidence bounds (dashed lines) for the tumor stages T1 and T2 to T3 (T2 and T3 in one group because only one single tumor of size T3 recurred)

Eight eyes (14.3%) showed a recurrence during the follow-up period, which included 6 (31.6%) eyes in group I and 2 (5.4%) group II. According to the AJCC Classification, recurrences occurred at T1 (*n* = 2, 0.8%), T2 (*n* = 5, 21.7%), and T3 (*n* = 1, 13.5%). Three patients were treated with a CCB and five with a CCD plaque. For the survival functions in Fig. 3, the log-rank test yielded *p* = 0.003 (χ2 = 8.929); the better progression-free survival in Group II was statistically significant. The curve fitting of a Cox proportional hazards function showed that the independent variable tumor location (Group I or Group II) was statistically significant with *p* = 0.011. The hazard ratio of Group II to Group I was 0.126 (within the bounds 0.027 and 0.599 for 95% confidence level) or 7.937 reciprocally. For the survival functions in Fig. 4, the log-rank test yielded *p* = 0.255 (χ2 = 1.296); there was no significant difference in progression-free survival between the tumor stages. The curve fitting of a Cox proportional hazards function showed that the predictor variable tumor stage (values T1 or T2, T3) was not statistically significant (*p* = 0.267). The hazard ratio of T2 and T3 to T1 was 2.478 (within the confidence bounds 0.615 and 9.960). The Cox proportional hazards model with both variables tumor location and tumor stage yielded the hazard ratios 0.133 and 2.117. The p values were 0.014 and 0.360; solely the variable tumor location adjusted for the tumor stage was statistically significant.

Overall, the local tumor control rate was 85.7% (48/56), particularly, 68.4% (13/19) in group I and 94.6% (35/37) in group II. After relapse, five of the eight eyes were further treated with fractionated stereotactic radiosurgery, and three eyes were enucleated. Recurrences were observed after 5–49 months, and the median time was 38.2 months. Three patients had their treated eyes enucleated because of high ocular pressure or hemorrhage. The overall eye-preservation rate was 89% (6/56), with 79% (4/19) in group I and 95% (2/37) in group II.

Distant metastasis was observed in seven cases (group I, *n* = 3; group II, *n* = 3) 14–100 months after brachytherapy. Nine patients died during follow-up (overall survival, 83.9%), and six died of distant disease progression resulting in a cancer-specific survival rate of 89.3%.

Endoresection as a post-therapeutic intervention was necessary in four patients (group I, *n* = 1; group II, *n* = 3) because of an increasing retinal detachment and to avoid a toxic tumor syndrome. Vitrectomy with silicone oil placement was performed in five eyes (group I, *n* = 2; group II, *n* = 3) because of persisting exudative retinal detachment after brachytherapy.

Radiation retinopathy was detected in 7 eyes (group I, 2/19, 11%; group II, 5/37, 14%), radiation optic neuropathy in 7 (group I, 5/19, 26%; group II, 2/37, 5%), radiation maculopathy in 10 (group I, 2/19, 11%; group II, 8/37, 21,6%), and macular edema in 6 (group I, *n* = 1; group II, *n* = 5). Macular edema was with intravitreal steroids treated in three cases and topical nonsteroidal anti-inflammatory eye drops (Nevanac®) in one case.

## Discussion

**Fig. 5:**
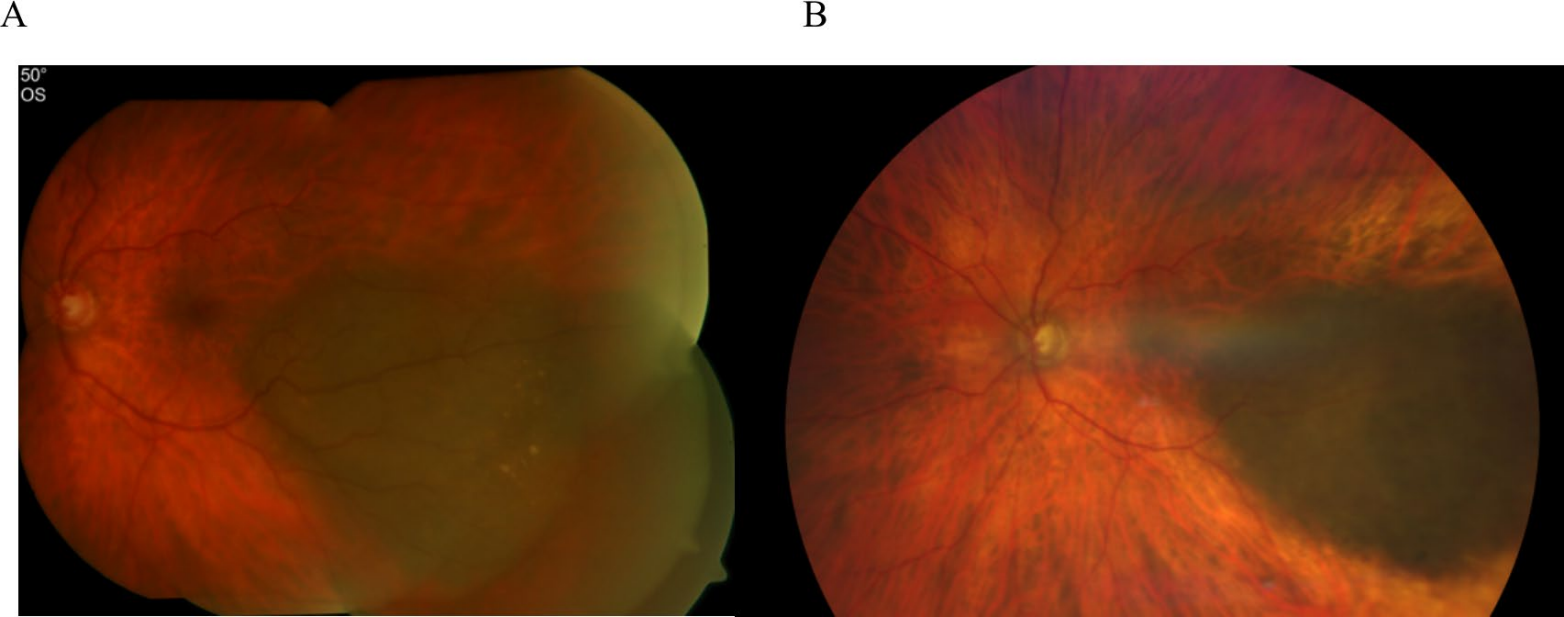
A case of a patient with a CM in group II: **a** VA 1.0; height, 2.4 mm. **b** 90 months after brachytherapy, VA 0.8; height, 1.4 mm

In this retrospective study, we showed that ruthenium plaque brachytherapy can be successfully used for the treatment for central UMs. The lateral or medial location to fovea affects the cure rates. Tumor size and localization are well established risk factors for local control. A localization close to the optic disc or center of the fovea has been associated with a higher recurrence rate [2]. Preserving the VA as best as possible may be challenging, the central border of the plaque covering the melanotic lesion while staying away from the fovea, which can be achieved if placing the plaque eccentrically. On the contrary, the optic disc is an anatomical limitation [18]. Figure 5 illustrates the fading tumor from the central structures 90 months after brachytherapy. Tumor thickness is a well-established limiting factor for brachytherapy. However, thickness was not a stringent exclusion criterion and used in selected cases included in the present series. Nine eyes with tumors larger than 6 mm were accepted. Patients with lesions exceeding 6 mm were treated after thorough discussion of alternative options and based on patients’ preferences. Two of them with UM larger than 7 mm in height were 87 and 80 years old and refused primary enucleation or external beam radiation therapy. Both were without disease progression for 35 and 23 months, respectively.

A meta-analysis by Karimi et al.*,* which included 21 peer-reviewed studies involving 3913 patients with UMs, showed similar results regarding the local tumor control rate of 84%, which ranged from 59 to 98% [10]. O´Day et al. reported treatment failure rate of 15.5% (34/219) in posterior UM [16], similar to the present series. In our series, the striking difference was LCR in respect of the primary tumor location was Tumor location. Indeed, the recurrence risk of central UMs varies from 69 to 100%, and a decreasing distance of the tumor margin to the optic disc up to < 2 mm has been associated with a higher recurrence rate [8, 9, 14, 18, 25]. Other treatment options such as proton beam or fractionated stereotactic photon beam radiotherapy achieve a higher 5 year overall local tumor control rate of 96.1% comparable to our results in group II [3].

Of the eight eyes with a recurrence, five were treated with salvage fractionated stereotactic radiosurgery, and three eyes were enucleated. No difference was found in the apex and scleral contact dosage regarding recurrence, localization, and enucleation. Three eyes had to be enucleated because of recurrence and three for circumstances such as high intraocular pressure and hemorrhage as radiation complications. Overall, our globe preservation rate was 89%. Similar to other studies, the globe retention rate was 95.4% [16], 84% at 5 years in juxtapapillary UM [19], and 89% for UM with macular involvement [9]. External beam radiation therapy such as proton beam or stereotactic radiosurgery has shown enucleation rates between 8 and 16% similar to those with brachytherapy [1, 12, 21].

Radiation complications such as retinopathy was detected in 7 (12.5%) eyes (group 1, 11%; group II, 14%), radiation optic neuropathy in 7 (12.5%) eyes (group I, 5 (26%); group II, 2 (5%)), and radiation maculopathy in 10 (17.9%) eyes (group I, 2 (11%); group II, 8 (21.6%)). Depending on the tumor height and distance to the optic disc and fovea, radiation complications vary. Russo et al. found radiation maculopathy in 7.4% and optic neuropathy in 1.8% of the cases. Sagoo et al*.* reported non-proliferative and proliferative radiation retinopathy in 66% and 24%, radiation optic neuropathy in 61%, and maculopathy in 56% after 5 years [18, 19]. Treatment of radiation complications includes laser coagulation, off-label intravitreal anti-vascular endothelial growth factor, and off-label intravitreal corticosteroids [28].

Radiation complications often cause a loss in VA. In addition, several studies have shown that the treatment of central tumors is associated with visual loss [4, 9, 17–19]. The mean presenting decimal VA in this study was 0.45 (group I, 0.37; group II, 0.49), and the final VA was 0.26 (group I, 0.15; group II, 0.32). Shields et al. showed that central UM < 5 mm from the optic disc or foveola had poor VA of ≤ 0.01 up to no light perception in 35% or 25% after 5 years [23].

In addition to brachytherapy, central UMs can also be treated with a proton beam (PBR) or fractionated stereotactic radiotherapy (fSRT). The first study that compared both treatment modalities did not find differences regarding the local control and adverse side effects. Vitreous hemorrhage may be more likely to occur in the group treated with stereotactic radiotherapy. No difference was also found concerning radiation maculopathy and optic neuropathy in central UMs [3]. The eccentric plaque placement in brachytherapy is an option to spare the fovea in the treatment and maintain a good VA. This can be promptly controlled through direct binocular fundoscopy while placing the plaque. Concerning all treatment options, an early detection of UMs and fast treatment are necessary for effective therapy because the UM size affects treatment success [3, 26].

In conclusion, despite the changes in central UM, this study shows that central UMs can be treated with Ru-106 plaques, providing local tumor control of 86% (group I 68%, group II 96%). Central UMs should be classified as lesions lateral or medial to the fovea achieving a statistically significant higher progression-free survival in the group located lateral to the fovea. E.g. adding information to the staging system might be sensible. Particularly, the localization near the optic disc (medially to the fovea, Group I) requires thoughtful management, and therapeutic options, such as external beam radiation therapy may be preferred. There was no significant difference in progression-free survival between the tumor stages.

We acknowledge the limitations of this study due to the short follow- up time of less than a year in one case. Moreover, the influence of the localization on local tumor control rate with regard to a further division into four quadrants, the temporal and nasal upper and temporal and nasal lower quadrants, have not yet been studied, and thus, requires further evaluation.

## Data Availability

No datasets were generated or analysed during the current study.
